# Serum Iron and Ferritin Levels in Beta Thalassemia Carriers in Duhok Governorate, Iraq

**DOI:** 10.7759/cureus.61748

**Published:** 2024-06-05

**Authors:** Aveen Mustafa

**Affiliations:** 1 College of Medicine, University of Duhok, Duhok, IRQ

**Keywords:** beta thalassemia trait, hba2, iron, ferritin, thalassemia

## Abstract

Background

Thalassemia and iron deficiency anemia (IDA) account for most cases of microcytic hypochromic anemia. It is a common misconception that iron deficiency does not occur in thalassemia. However, studies have found that iron deficiency can coexist in carriers of beta thalassemia.

Objective

The aim of this study was to determine the prevalence of iron deficiency and iron overload in carriers of beta thalassemia in Duhok, Iraq.

Patients and methods

This prospective cross-sectional study included 250 patients with beta thalassemia carriers attending Kurdistan Private Hospital Laboratory Department from July 2021 to June 2023. Patients with microcytic hypochromic blood picture were tested for HbA2 levels, and those with a level >3.7% were included in the study and were tested for serum iron and serum ferritin levels.

Results

The age range was 15-80 years, with a mean of 25 years, and the male-to-female ratio was 1.5:1. The prevalence of iron deficiency in beta thalassemia carriers was 16% (N = 40). The prevalence of iron overload was 8.4% (N = 21). There was a significant statistical difference among those with iron deficiency, normal iron status, and iron overload in terms of hemoglobin level (*P*=0.001), RBC count (*P*=0.012), HbA2 (*P*=0.015), and serum ferritin (*P* < 0.0001).

Conclusion

Iron deficiency is more prevalent in beta thalassemia carriers than iron overload, necessitating proper assessment of iron status in patients with beta thalassemia carriers. Those with abnormal iron status need effective treatment to optimize the overall outcomes in patients with beta thalassemia carriers.

## Introduction

Iron deficiency anemia (IDA) and thalassemia are the two most frequently encountered causes of microcytic hypochromic anemia [1]. Thalassemia is one of the most common worldwide genetic diseases, with the World Health Organization (WHO) estimating a global carrier rate of around 7%. Its prevalence is high in the Mediterranean area and the Middle East, with estimated carrier rates approaching 30% [2]. Thalassemia syndromes are inherited as autosomal recessive disorders, resulting in defective globin synthesis. They can manifest in a spectrum ranging from asymptomatic carriers to major thalassemia that requires frequent blood transfusion [3]. Thalassemia trait is a carrier status developing from single allele mutation [4].

Anemia is the most common blood disorder, with estimates suggesting that around one-third of the world’s population have anemia, mostly IDA [5]. Despite the declining prevalence of IDA globally, iron deficiency continues to be at the top of the etiology list for IDA. The terms anemia, iron deficiency, and IDA are used interchangeably but they are not equivalent. In iron deficiency, the iron stores are reduced, and this can progress to IDA or persist without progression. In IDA, the iron stores are more depleted, and this results in anemia with a microcytic hypochromic blood picture [6]. The diagnosis of iron deficiency status has been suggested by many tests over the years, but serum ferritin is nowadays the most cost-effective, efficient, sensitive, and specific test, given the shortcomings of other tests (indicated by a level of <30 μg/L). Ferritin levels are lower in IDA [6-8].

The concurrence of iron deficiency and thalassemia has been a debatable subject with the existence of conflicting data about iron metabolism in thalassemia [9]. It has long been assumed that iron deficiency does not occur in thalassemia syndromes. However, studies have revealed the occurrence of iron deficiency in thalassemia carriers, with hemoglobin levels being lower in those with concurrent IDA and thalassemia carriers [1,3]. The existence of iron deficiency in thalassemia carriers is often overlooked. It is very difficult to distinguish IDA from thalassemia carriers with certainty based on clinical manifestation or blood picture or indices [2]. This distinction is critical as ignoring this simultaneous prevalence can perpetuate anemia, making it severe enough to affect the overall condition of the patient [4].

The objective of this study was to measure the prevalence of iron deficiency and iron overload in carriers of beta thalassemia, which would give an insight into the size of the problem in our locality and guide the treating physicians in decision-making regarding iron therapy.

## Materials and methods

This prospective cross-sectional study included patients attending the Kurdistan Private Hospital Laboratory Department in Duhok, Kurdistan Region of Iraq (KRG), over a period of two years, from July 2021 to June 2023. The study objective was explained to the patients, and informed consent was obtained. Patients with microcytic hypochromic blood picture were tested for HbA2 levels using high-performance liquid chromatography (HPLC), and those with a level 3.7% and above were included in the study. This threshold is based on established diagnostic criteria for beta thalassemia carriers. The precise threshold can vary, with some studies setting the cut-off at 3.7% or 3.8% [5]. Exclusion criteria were patients with febrile illnesses, those with suspected inflammatory disorders, use of iron supplements, and those with other hemoglobinopathies.

For patients who were enrolled in the study, the serum sample was frozen at -20 ºC for the measurement of serum iron (by Cobas c-111) and ferritin level (by a Cobas e-411). Serum ferritin <30 μg/L was used to define iron deficiency [6-8], while iron overload was defined as serum ferritin >300 μg/L [10-12]. One limitation of our study was the insufficient serum volume to measure total iron-binding capacity (TIBC) for all participants, which would have allowed for the calculation of transferrin saturation, providing a more comprehensive assessment of iron status. The normal serum iron level ranges of the manufacturer are 60-190 μg/dL for males and 40-175 μg/dL for females [13]. The normal hemoglobin ranges are 14-18 g/dL for males and 12-16 g/dL for females [14]. The normal range for mean corpuscular volume (MCV) is 80-100 fL, and that for mean corpuscular hemoglobin (MCH) is 27-33 pg. Anemia is defined as a hemoglobin level below 13 g/dL in males and below 12 g/dL in females, according to the WHO criteria. These normal ranges were used to determine microcytic hypochromic anemia in all patients included in the study.

Data tabulation and analysis were performed using the Statistical Package for the Social Sciences (SPSS) program Version 28.0.1 (IBM Corp., Armonk, NY). The P-value for categorical data was calculated using the chi-square (χ²) test, while analysis of variance (ANOVA) was used to test for differences among the means of groups. The statistical associations or differences were considered significant if the P-value was less than 0.05.

## Results

The total sample size was 250 cases. Male cases were 150, and female cases were 100 (male-to-female ratio = 1.5:1). The mean age of the patients was 25 years (standard deviation = 7.8 years), and the age range was 15-80 years. The most common age group was 20-30 years (N=146, 58.4%), followed by 10-20 years (N=56, 22.4%) and 30-40 years (N=35, 14%), with only 13 (5.2%) cases being older than 40 years. The laboratory data of the cases are presented in Table 1, including the complete blood count and hemoglobin electrophoresis parameters, in addition to serum iron and serum ferritin.

**Table 1:** Laboratory data of the study patients (N =250) RBC, red blood cell; MCV, mean corpuscular volume; MCH, mean corpuscular hemoglobin

Parameters	Range	Mean	Standard deviation
Hemoglobin (g/dL)	7.1-17.5	12.4	1.4
RBC count × 10^12^	3.3-7.6	6.1	0.6
MCV (fL)	48-77	62.6	4.9
MCH (pg)	16.9-25.6	20.5	1.6
HbA2 (%)	3.7-9.1	5.51	0.89
Serum iron (μg/dL)	17-387	150	59.3
Serum ferritin (μg/L)	5-1,155	135	144

The prevalence of iron deficiency in beta thalassemia carriers was 16% (N = 40), with nine cases being male and 31 cases being female. The prevalence of iron overload was 8.4% (N = 21), with 20 cases being males (95.5%) and only one case being female (4.5%). There was a significant statistical difference between males and females regarding iron deficiency and iron overload, with P-value being < 0.001 in both statuses. Of the study patients, 71.6% were anemic (N=179) with no significant statistical difference regarding those with iron deficiency, normal iron status, and iron overload (P = 0.7). The serum iron level was lower than normal range in only two females and three males (2%), while it was higher than normal range in 17 females (17%) and 40 males (27%).

 The statistical testing for differences among those with iron deficiency, normal iron status, and iron overload is shown in Table 2. There was a significant statistical difference regarding hemoglobin level (P=0.001), RBC count (P=0.012), HbA2 (P=0.015), and serum ferritin (P < 0.0001). There was no significant statistical difference regarding age, MCV, MCH, red cell distribution width (RDW), and serum iron (P ≥ 0.05).

**Table 2:** Comparison between subjects regarding iron status (N =250) RBC, red blood cell; MCV, mean corpuscular volume; MCH, mean corpuscular hemoglobin; RDW, red cell distribution width

Parameters	Iron deficiency (N =40), mean ± SD	Normal iron status (N = 189), mean ± SD	Iron overload (N = 21), mean ± SD	P-value
Age	23.7 ± 4.6	26.1 ± 8.4	26.9 ± 6.4	0.16
Hemoglobin (g/dL)	11.7 ± 1.4	12.6 ± 1.5	12.8 ± 1.2	0.001
RBC count × 10^12^	5.8 ± 0.7	6.1 ± 0.6	6.2 ± 0.6	0.012
MCV (fL)	62.2 ± 5	62.7 ± 4.9	62.9 ± 4.8	0.8
MCH (pg)	20.2 ± 1.6	20.6 ± 1.6	20.7 ± 1.4	0.31
RDW (%)	13.9 ± 2.1	13.7 ± 1.8	13.9 ± 1.5	0.75
HbA2 (%)	5.1 ± 0.9	5.5 ± 0.9	5.8 ± 0.7	0.015
Serum iron (μg/dL)	144 ± 54	146 ± 76	183 ± 79	0.08
Serum ferritin (μg/L)	17.9 ± 6.2	120 ± 76	484 ± 205	< 0.0001

## Discussion

Beta thalassemia is common in the Mediterranean area [7] and has a carrier rate of 3.7-6.9% [15] in Northern Iraq, with a previous study revealing the carrier rate to be 3.7% in Duhok governorate [16]. Data on iron metabolism in beta thalassemia carriers have shown conflicting findings. Beta thalassemia carriers can lead to mild ineffective erythropoiesis complicated by exaggerated iron absorption, which is expected to protect the patient from iron deficiency, but this has not been reproduced consistently in all the studies. Some studies revealed normal iron stores reflected by serum ferritin level measurement, while others have found that they frequently have positive iron balance that put them at a high risk for iron overload, with the harm being increased if these patients are given iron therapy. Conversely, other studies have shown a higher prevalence of iron deficiency in thalassemia carriers than in general patients [2]. These differing findings have increased my interest in exploring the situation in our area.

The prevalence of iron deficiency in thalassemia carriers was 16%, which is lower than the figures observed by Dolai et al. (19.3%) [3], Hasan (34.6%) [17], Karnpean et al. (20.4%) [18], and Rahman et al. (30.2%) [2]. The prevalence of iron overload was 8.4%, with 95.5% being males and only 4.5% being females, which is close to the rate reported by Yousafzai et al. (10%) [19]. Anemia was present in 71.6% of the study patients, which is lower than that reported by Hasan (88.3%) [17].

The causes of iron deficiency are insufficient dietary intake, chronic blood loss, and/or malabsorption. The lower rates of iron deficiency and anemia reported in this study can be explained by the fact that the prevalence of IDA is generally decreasing in developing countries, attributed to improved nutritional status [6]. On the other hand, the prevalence rate of iron deficiency at 16% is higher than the rate of iron overload (8.4%), and this is very important to consider in patients with thalassemia carriers, as many clinicians think that thalassemia puts the patient at positive iron balance and ask patients to avoid iron-rich foods and supplements aiming at reducing the burden of iron overload. This attitude can lead to IDA, which can complicate the already existing anemia from ineffective erythropoiesis [17]. Iron overload was more common in males, as most of the patients in this study were in the reproductive stage in which menstruation can protect the females from iron overload [19].

The mean hemoglobin level and red blood cell count were lower in those with iron deficiency than in those with normal iron status, which is consistent with findings from other studies [17,19]. MCV and MCH were also lower in those with iron deficiency. The fact that the blood picture in thalassemia carriers is microcytic hypochromic should not be attributed solely to defective erythropoiesis; there may be associated iron deficiency that warrants correction to increase the hemoglobin level and reduce the adverse consequences of anemia in these patients [2].

The RDW was higher in those with iron deficiency than those with normal iron status, which is consistent with the findings of other studies [18,19]. This index indicates the degree of RBC anisocytosis and is helpful in differentiating IDA from thalassemia carriers [20]. The HbA2 levels were lower in iron deficiency than those with normal iron status and iron overload. This is consistent with the results observed by Yousafzai et al. [19] but contrary to the results observed by Hasan [17]. HbA2 measurement has a pivotal role in beta thalassemia screening programs. Some studies have shown that iron deficiency results in reduced synthesis of HbA2, which could result in normal HbA2 levels, although other studies have refuted this assumption; however, it is important to screen for and correct any iron deficiency before beta thalassemia screening is undertaken [21].

There were, however, some limitations to our study that needed to be addressed. To start with, even though this study took a significant sample size of 250 patients, it may not be representative of the general population in Dohuk about beta thalassemia carriers, and thus the results may not be generalizable. Secondly, the cross-sectional design in this study could only give a picture of the iron status in this patient group at the time of sampling, without taking into account the risks of possible fluctuation of iron over time or seasonality variation that might affect iron metabolism. Third, even though serum ferritin and serum iron are widely used and accepted markers of the body's iron content, we did not take into account the influences of acute phase reactions or other inflammatory conditions that could cause independent elevations in ferritin. Consequently, the results could still be influenced by subclinical inflammation. In addition, the exclusion criteria of febrile illnesses and the use of iron supplementation, although necessary to eliminate some of the confounders, might have eliminated respondents who could represent a greater proportion of our target population. Finally, this study did not evaluate possible genetic variations in iron metabolism among the participants that differently affect iron absorption and storage among beta thalassemia carriers. Thus, long-term longitudinal studies with larger and more diversified cohorts are necessary to confirm these findings and investigate the underlying mechanisms of these changes in greater detail. Recognition of these limitations is important with respect to the drawing of the results and for designing future studies to fine-tune the management of iron status in beta thalassemia carriers.

## Conclusions

We conclude that iron deficiency is more prevalent in beta thalassemia trait than iron overload and that it is important to conduct proper assessment of iron status in beta thalassemia trait. In case of iron deficiency, it is important to treat it to reduce the overall burden of anemia in these patients.
